# Protein profiles of hatchery egg shell membrane

**DOI:** 10.1186/s12953-017-0112-6

**Published:** 2017-03-03

**Authors:** N. C. Rath, R. Liyanage, S. K. Makkar, J. O. Lay

**Affiliations:** 1 USDA/Agricultural Research Service, Poultry Production and Product Safety Research Unit, Fayetteville, AR 72701 USA; 2 Statewide Mass Spectrometry Facility, University of Arkansas, Fayetteville, AR 72701 USA; 3 Department of Poultry Science, University of Arkansas, Fayetteville, AR 72701 USA

**Keywords:** Hatchery egg shell membrane, Proteins, Mass spectrometry

## Abstract

**Background:**

Eggshells which consist largely of calcareous outer shell and shell membranes, constitute a significant part of poultry hatchery waste. The shell membranes (ESM) not only contain proteins that originate from egg whites but also from the developing embryos and different contaminants of microbial and environmental origins. As feed supplements, during post hatch growth, the hatchery egg shell membranes (HESM) have shown potential for imparting resistance of chickens to endotoxin stress and exert positive health effects. Considering that these effects are mediated by the bioactive proteins and peptides present in the membrane, the objective of the study was to identify the protein profiles of hatchery eggshell membranes (HESM).

**Methods:**

Hatchery egg shell membranes were extracted with acidified methanol and a guanidine hydrochloride buffer then subjected to reduction/alkylation, and trypsin digestion. The methanol extract was additionally analyzed by matrix assisted laser desorption ionization-time of flight mass spectrometry (MALDI-TOF-MS). The tryptic digests were analyzed by liquid chromatography and tandem mass spectrometry (LC-MS-MS) to identify the proteins.

**Results:**

Our results showed the presence of several proteins that are inherent and abundant in egg white such as, ovalbumin, ovotransferrin, ovocleidin-116, and lysozyme, and several proteins associated with cytoskeletal, cell signaling, antimicrobial, and catalytic functions involving carbohydrate, nucleic acid, and protein metabolisms. There were some blood derived proteins most likely originating from the embryos and several other proteins identified with different aerobic, anaerobic, gram positive, gram negative, soil, and marine bacterial species some commensals and others zoonotic.

**Conclusion:**

The variety of bioactive proteins, particularly the cell signaling and enzymatic proteins along with the diverse microbial proteins, make the HESM suitable for nutritional and biological application to improve post hatch immunity of poultry.

**Electronic supplementary material:**

The online version of this article (doi:10.1186/s12953-017-0112-6) contains supplementary material, which is available to authorized users.

## Background

Egg shells that constitute a significant part of poultry hatchery waste, comprise largely of calcium carbonate crusts and underlying shell membranes laced with proteins originating from egg whites as well as contaminants of microbial and environmental origins [1]. The shell membranes have been considered for various engineering, agricultural, and biomedical applications, spurring many studies of their biochemical compositions [2–9]. Most such studies however, have used membranes harvested from fresh eggs and shown to contain proteins and peptides of structural, antimicrobial, cell regulatory, and stress protective categories which likely contribute to their some of their health beneficial and therapeutic effects [2, 10–19]. Recently, we reported that the hatchery shell membranes (HESM), used as nutritional supplement, in post hatch chicks, promote growth, show protection against bacterial lipopolysaccharide (LPS) induced inflammatory response and diminish stress markers such as plasma levels of corticosterone [20]. Presuming that these effects could be mediated through the bioactive proteins present in the HESM, we were interested to analyze the proteomic composition of HESM that has never been investigated. Besides, the protein population of HESM can be qualitatively different from the fresh egg shell membrane because the embryo development not only induce dynamic changes in egg proteome [21, 22] but also, alter the membrane structure to facilitate the hatching process. Hence, the current study was conducted utilizing the HESM that were dried at room temperature in a chemical hood without additional cleanup for 2 month period in the room temperature.

## Methods

### Chemicals and reagents

The reagents and devices such as C18 Nu tips (Glysci.com), Spectra/Por membranes (Spectrumlabs.com), bicinchoninic acid (BCA) protein assay kit, Pierce C18 spin columns, MS grade trypsin (Fisher Scientific.com), peptide and protein calibration standards (*m/z* 500–16000, Bruker Daltonics, Bremen, Germany), and 2-iodoacetamide (IAA) (MP Biomedical, OH) were purchased from their respective vendors. Most other chemicals and supplies including 1, 4-dithiothreitol (DTT), 2, 5-dihydroxybenzoic acid (DHB), were purchased from Sigma Aldrich (St. Louis, MO).

### HESM preparation

Empty eggshells were obtained from the University of Arkansas poultry hatcheries, dried at room temperature under the hood for 2 months, the membranes separated manually then ground to coarse powder using an IKA mill (Cole Palmer). Duplicate samples of membrane powder from two separate preparations, each made from >50 eggs, were extracted (a) with 0.1% acetic acidified 70% methanol for soluble proteins, peptides, and their degradation products, and (b) with 4 M guanidine hydrochloride (GuHCl) containing 20 mM EDTA, and 50 mM Na-acetate, pH 5.8 for all soluble proteins [17, 23]. The membrane powder were extracted with 20 volumes of respective solutions overnight at 4 °C by constant stirring then centrifuged at 20,000 g for 15 min and the supernatants containing all soluble factors were dialyzed against excess volumes of 50 mM ammonium bicarbonate with three successive changes using 1000 Da Spectra/Por dialysis membranes. The solutions containing approximately 20 μg of protein from each extract, measured by BCA protein assay, were reduced with 10 mM dithiothreitol (DTT) for 1 h at 60 °C and alkylated with 40 mM iodoacetamide (IAA) then digested with 0.4 μg trypsin for 48 h at 37 °C [23, 24]. The tryptic digests were desalted using Pierce C18 spin columns according to the manufacturer suggested protocol prior to liquid chromatography-tandem mass spectrometry (LC-MS/MS).

### LC-MS/MS analysis

Following desalting with C18 columns, the peptides were eluted, dried and re-suspended in 0.1% formic acid (FA) then subjected to LC-MS/MS using an Agilent 1200 series micro flow HPLC coupled to a Bruker Amazon-SL quadrupole ion trap mass spectrometer, and a captive spray ionization source. Tryptic peptides were separated at a solvent flow rate of 1.6 μL/min with 0 to 40% gradients of 0.1% FA (solvent A) and acetonitrile (ACN) in 0.1% FA (solvent B) over a period of 300 min each using a C_18_ (150 × 0.1 mm, 3.5 μm particle size, 300 Å pore size, Zorbax SB) capillary column. The captive electrospray source was operated in a positive ion mode with a dry gas temperature of 150 °C, dry nitrogen flow 3 L/minute, and capillary voltage of 1500 V. The data were acquired in the auto MS (*n*) mode with optimized trapping condition for the ions at *m*/*z* 1000. MS scans were performed in the enhanced scanning mode (8100 *m*/*z*/second), while the collision-induced dissociation or the MS/MS fragmentation scans performed automatically for top ten precursor ions for 1 min each in the UltraScan mode (32,500 *m*/*z*/second). The samples were run three times as technical repeats.

### MALDI-TOF analysis

For direct matrix assisted laser desorption ionization-time of flight mass spectrometry (MALDI-TOF-MS), aliquots of methanol extracts were subjected to reduction/ alkylation using DTT and iodoacetamide (IAA) as described previously [25] or mock treated omitting DTT from the reaction for respective controls then spotted on a MALDI target plate mixing at 1:1 ratio with two dihydroxybenzoic acid. In all cases, samples were first subjected C18 nu tip cleaning up process using manufacturer recommended protocol before spotting on the MALDI target plate. The mass spectra were acquired with a Bruker Ultraflex II MALDI-TOF mass spectrometer (Bruker Daltonics GMBH, Bremen, Germany) operated in the positive-ion reflectron mode with the TOF analyzer calibrated with Bruker peptide standard II. Accurate mono isotopic protonated, intact peptide, masses were determined by MALDI-TOF-MS using combinations of external and internal calibration procedures, spotting the samples with equal volumes of α-cyano-4-hydroxycinnamic acid matrix, prepared in 0.1% FA, and 50:50 water/ACN. The MALDI-TOF-MS data were processed and some selective peaks were fragmented using LIFT-TOF/TOF MS to obtain their identities [17].

### Data analysis

Peaks were picked from the LC-MS/MS chromatogram using instrument’s default settings and the mzML files created using Bruker Data Analysis 4.0 software. The mzML files created as such were used in Bruker ProteinScape 3.1 server with MASCOT data base search tool to perform MS/MS data search against the UniProt Gallus and NCBI bacterial data bases to identify the proteins. All proteins were identified with a 95% confidence limit and <1% false discovery rate with at least one unique peptide. The parent ion mass- and fragment ion mass tolerance were both, set at 0.6 Da with cysteine carbamidomethylation and methionine oxidation as fixed and variable modifications. MASCOT.dat data base search files and.mzML raw data files were then exported into Skyline v3.1 software (http://proteome.gs.washington.edu/software/Skyline) [26, 27] to further refine the tryptic peptide identification based on their relative hydrophobicity and retention time correlations. Protein identities were also refined based on the uniqueness of the identified tryptic peptides by comparing them against respective background proteomes (either UniProt Gallus or the NCBI Bacteria data bases). The MALDI-TOF-MS data were processed using Bruker Flex Analysis 3.3 and Bruker BioTools 3.1 software. Peptides were identified by MASCOT MS/MS ion search tool with a peptide mass tolerance of 200 ppm and MS/MS ion tolerance of 0.6 Da. Functional annotation and classification of the proteins identified by LC-MS/MS with two or more unique peptides were performed using gene ontology (GO) based PANTHER classification system (http://www.pantherdb.org) [28] and proteins refined by Skyline from both guanidine and methanol extracts. Also, the same proteins were used in STRING functional protein association networks, version 10 (http://string-db.org) [29], under a high confidence setting to identify protein co-occurrence and possible functional relations.

## Results

**Fig. 1:**
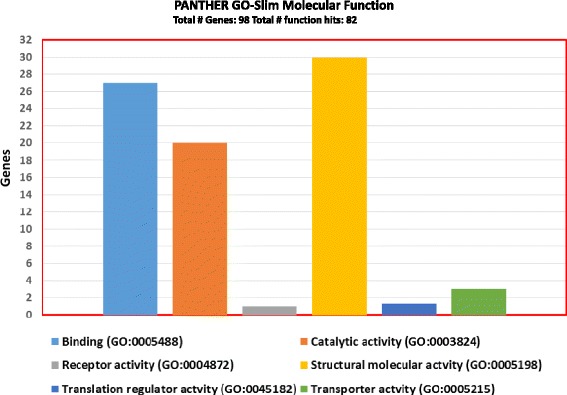
Molecular function annotation of proteins, identified with two or more unique peptides, using Protein Analysis through Evolutionary Relationships (PANTHER)

**Fig. 2:**
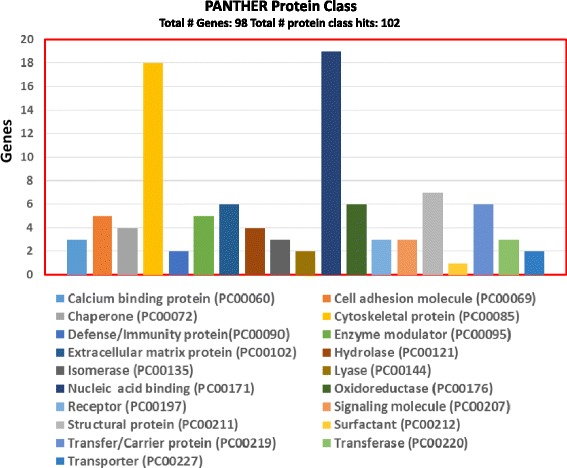
Functional annotation by protein class of HESM proteins, identified with two or more unique peptides, using Protein Analysis through Evolutionary Relationships (PANTHER)

**Fig. 3:**
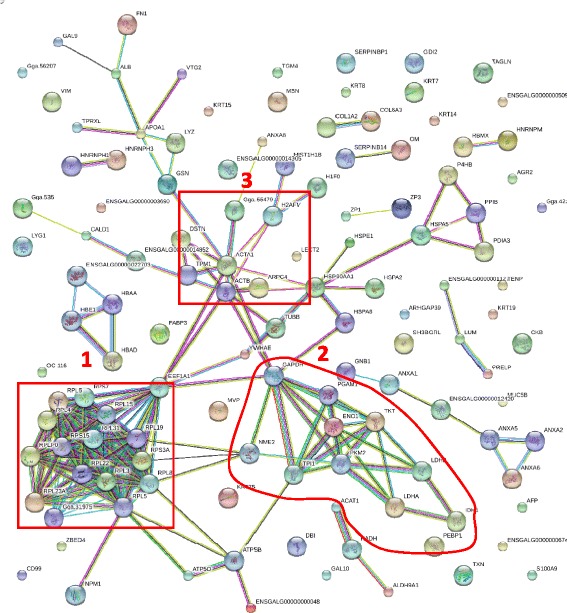
The protein-protein interaction network of proteins identified with two or more unique peptides. Rectangles showing, 1. Actin node, 2. Ubiquitin node, and 3. Ribosomal and nuclear protein clusters

**Fig. 4:**
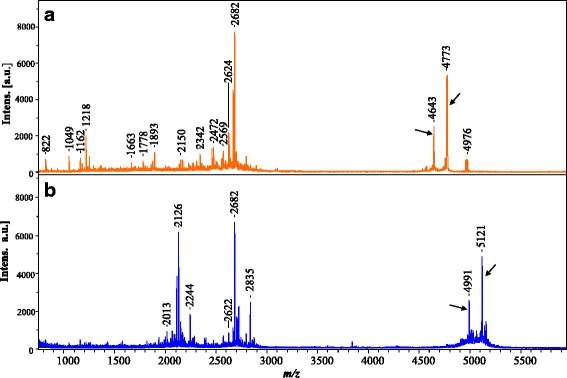
MALDI-TOF profile of HESM methanol extract before (**a**) and after reduction and alkylation (**b**). Two peaks corresponding to m/z 4643 and 4773 (**a**) show mass shifts by 348 Da corresponding to m/z 4991 and 5121 (**b**), respectively (*arrows*)

**Fig. 5:**
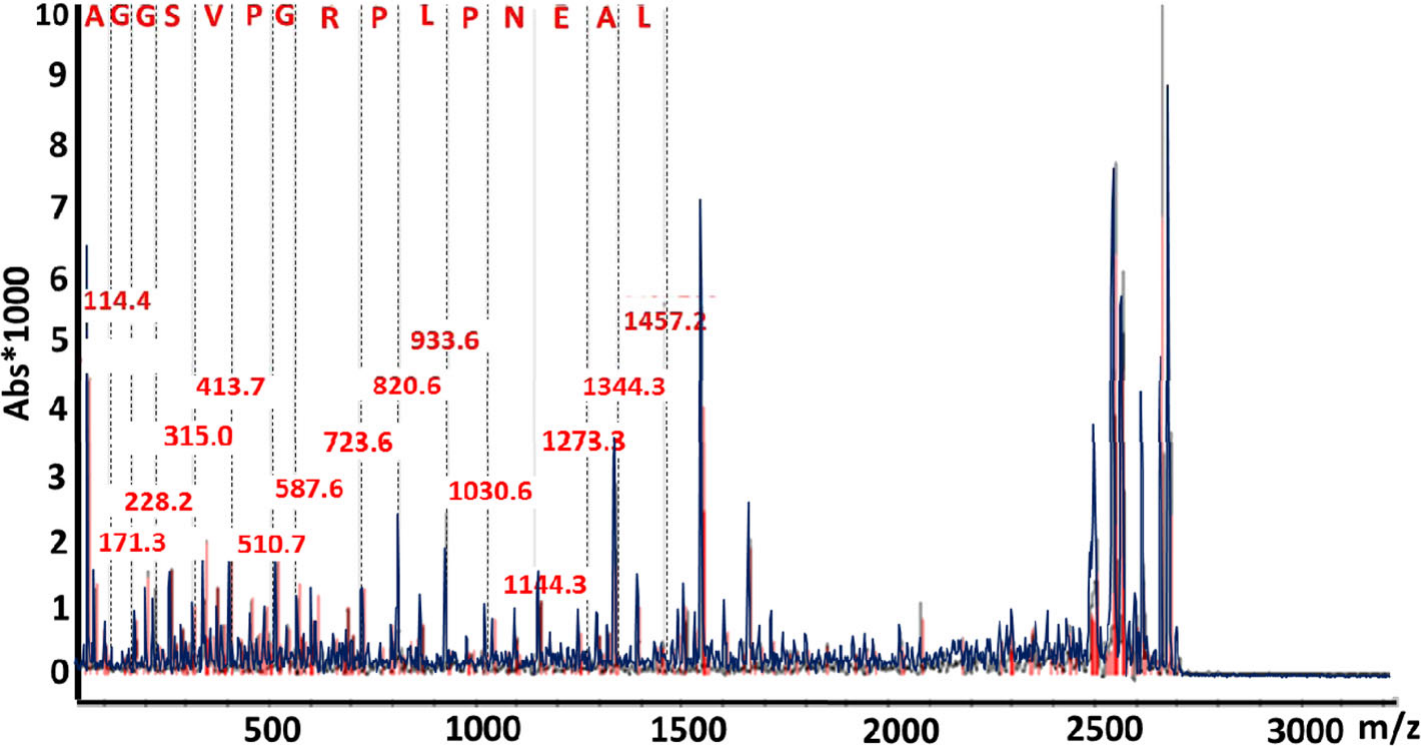
Fragmentation of m/z 2682 showing a partial sequence ‘AGGSVPGRPLPNEAL’ corresponding to Eimeria Apicomplexan specific protein matching by >92% identity

Table 1 shows the list of 41 proteins identified in the methanol extract using LC-MS/MS analyses and the Additional file 1: Table S1 shows the identities of 167 proteins in the GuHCl extracts of which 11 were common in both extracts which occur in egg white such as ovalbumin, ovomucoid, ovocleidin-116, and lysozyme (Table 1, names underlined). Several identified proteins on the basis of a single peptide were considered tentative although they were identified repeatedly in replicate samples and by Skyline retention time correlation refinements. These identifications were largely occurred in methanol extract which may relate to degraded proteins. There were 12 uncharacterized proteins including one in methanol extract. Several structural proteins that included collagens, keratin, proteoglycans (lumican, decorin) were identified in the HESM. Different keratins that included both cytoskeletal type 1 and non-cytoskeletal were abundant in HESM. Many cytoskeletal and their cognate proteins such as actin, vinculin, gelsolin, tubulin, vimentin, thymosin β4, and some blood associated proteins (annexins, hemoglobin, epsilon globin, and serum albumin) predominated in the guanidine extract. Cell signaling and enzyme proteins largely, associated with energy, protein, and nucleic acid metabolisms, and several antimicrobial proteins that included ovotransferrin, lysozyme, ovocleidin-116, ovocalyxin-36, keratin peptides, and gallinacin 9 and 10, were present. Some of the other proteins that secured high scores were embryonic and development associated proteins such as zona pellucida sperm binding proteins, elongation factors, and histone cluster proteins. Functional annotation with PANTHER using combined protein IDs from both extracts (methanol and guanidine) and GO-slim molecular function sorted the proteins into six major categories using 98 gene products with the binding, structural, and catalytic proteins topping the list (Fig. 1). A classification of protein functionality showed two dominant associations, one, with nucleic acid binding and the other with cytoskeletal function, followed by structural, transfer/ carrier, and oxidoreductase genre of proteins (Fig. 2). Some proteins uniquely present in methanol extract of HESM, such as gallinacin 9, thymosin β4, and septin, were not detected in the guanidine HCl extract. A protein interaction and co-occurrence map, using STRING bioinformatics, showed a major cluster of proteins associated with nuclear and metabolic activities (eg: ribosomal proteins) and two others associated with carbohydrate metabolism and actin/ actin-cognate function, respectively (Fig. 3). The MALDI-TOF MS of methanol extract showed several peaks of which 2, corresponding to *m/z* 4643 and 4773 (Fig. 4a), showed mass shifts by 348 Da upon reduction/ alkylation (Fig. 4b), suggestive of carbamidomethylation of three disulfides, whereas, few other peaks such as *m/z* 2682, 2624, 2835, and 2126 did not show any change. Based on our previous identification in ESM [17], we surmised that the *m/z* 4773 was the mature gallinacin 10 peptide with a corresponding sequence of “DPLFPDTVACRTQGNFCRAGACPPTFTISGQCHGGLLNCCAKIPAQ” and the *m/z* 4643 was the same peptide with the truncation of glutamine (Q) from the N-terminal. Fragmentation of *m/z* 2682 peptide by LIFT-MS/MS (Fig. 5) yielded a partial sequence corresponding to “AGGSVPGRPLPNEAL” which upon protein blast (BLASTP) (http://blast.ncbi.nlm.nih.gov/Blast.cgi?PAGE=Proteins), in non-redundant protein data base showed >90% identity with one Apicomplexan specific protein associated with Eimeria, a protozoa that causes coccidiosis in poultry. Using NCBI bacteria data base search, the proteins in GuHCl extract, showed 50 proteins belonging to different species of gram positive, gram negative, aerobic and anaerobic families of bacteria that included Candidatus, Enterococci, Yersinia, and Butyrivibrio, and several phytobacteria (Sphingomonas *taxi*), Nocardioide (soil bacteria), and a marine bacterium (hyphomonas, marinobacter adhaerens) (Additional file 1: Table S2). Many of these identifications were tentative based on the matches with their respective enzyme proteins with one unique peptide occurring in replicate sample. The maximum number of bacterial proteins identified belonged to Pseudomonas M10, a family of gram negative bacteria followed by Candidatus, Hyphomonas, and Sphingomonas.

**Table 1:** Proteins identified in methanol extract by LC-MS/MS. Proteins with a single peptide are considered “tentatively identified”

	Accession	Protein	MW [kDa]	Scores	# Peptides
1	ENSGALP00000036403	Ovalbumin	42.9	629.9	11
2	ENSGALP00000005544	Ovomucoid	22.6	338.1	7
3	ENSGALP00000017755	Ovocleidin-116	76.8	225	2
4	ENSGALP00000008163	Orosomucoid 1 (ovoglycoprotein) precursor	22.3	167	3
5	ENSGALP00000042635	peptidyl-prolyl cis-trans isomerase FKBP1A	8.9	163.5	3
6	ENSGALP00000016177	Lysozyme C	16.2	129.2	2
7	ENSGALP00000016632	keratin 8, type II	42.1	119.1	2
8	ENSGALP00000031725	SH3 domain binding glutamic acid-rich protein like (SH3BGRL), mRNA	12.9	109.6	2
9	ENSGALP00000027483	Ubiquitin-fold modifier 1	9	102.1	1
10	ENSGALP00000035930	Gallinacin-9	7.3	85.7	2
11	ENSGALP00000010763	Ovocalyxin-36 precursor	58.3	83.7	1
12	ENSGALP00000019758	Diazepam binding inhibitor (GABA receptor modulator, acyl-CoA binding protein) (DBI), mRNA.	9.6	69.8	2
13	ENSGALP00000026846	Gallinacin-10	7.1	62.6	2
14	ENSGALP00000006093	Keratin, type I cytoskeletal 19	46	62.3	3
15	ENSGALP00000009976	Usher syndrome 1C	100.1	51.9	1
16	ENSGALP00000012729	Uncharacterized protein	21	50.9	1
17	ENSGALP00000040654	Thymosin, beta 4	5	47.8	2
18	ENSGALP00000043411	Collagen, type XVI, alpha 1	156.6	42	1
19	ENSGALP00000002523	Signal peptidase complex subunit 1 homolog	27.2	37.9	1
20	ENSGALP00000013908	Zinc finger BED domain-containing protein 4	132.4	37.8	2
21	ENSGALP00000038912	Alpha-D-globin (HBAD), mRNA.	15.7	37.3	1
22	ENSGALP00000019988	Utrophin	398.6	36.5	2
23	ENSGALP00000000876	Fatty acid-binding protein, heart	14.8	34.6	1
24	ENSGALP00000026777	Nociceptin precursor	21.4	34.4	1
25	ENSGALP00000026863	Polycystic kidney and hepatic disease 1 (autosomal recessive)	440	32.7	1
26	ENSGALP00000018601	Serine peptidase inhibitor, Kazal type 2 (acrosin-trypsin inhibitor)	6	26.7	1
27	ENSGALP00000025439	Probable arginyl-tRNA synthetase, mitochondrial	65.3	25.2	1
28	ENSGALP00000039913	Transforming, acidic coiled-coil containing protein 1	86.4	24.5	2
29	ENSMGAP00000001722	elaC ribonuclease Z 2	94.2	24.2	1
30	ENSGALP00000031519	Fibroblast growth factor 2	16.2	22.8	1
31	ENSGALP00000039675	Nuclear receptor coactivator 2	44.7	21.5	1
32	ENSGALP00000000325	WD repeat-containing protein 36	98.2	21.2	1
33	ENSGALP00000019412	Septin 3	40	20.5	1
34	ENSGALP00000027541	High mobility group protein B1	24.9	20	1
35	ENSGALP00000014919	tRNA (adenine-N(1)-)-methyltransferase non-catalytic subunit TRM6	54.2	19.8	1
36	ENSGALP00000043133	Fibrinogen silencer binding protein	36.4	19	1
37	ENSGALP00000000726	SH3 domain binding glutamate-rich protein like 3	10.5	18.8	1
38	ENSGALP00000038283	UPF3 regulator of nonsense transcripts homolog B (yeast)	56.9	18.4	1
39	ENSGALP00000000275	Mutated in colorectal cancers	112.6	18.3	1
40	ENSGALP00000038735	Polyubiquitin-B Ubiquitin	109.6	15.5	1
41	ENSGALP00000040476	Neuregulin 2	65.5	15	1

The underlined proteins also occur in guanidine HCl extract (Additional file 1: Table S1)

## Discussion

As expected the HESM contained many proteins such as ovalbumin, ovomucoid, ovocleidin-116, and lysozyme that are inherent to egg proteome [8, 30–32]. Structural proteins such as keratins, collagens, decorin, lumican, and tropomyosin, and cytoskeletal proteins, such as actin, tubulin, vimentin, and their interacting proteins, were present along with several blood (hemoglobin, α-D-globin, annexin, fibronectin) and embryo associated proteins (vitellogenin, zona pellucida sperm-binding proteins, and vitelline membrane outer layer protein). These results are consistent with the reports of Cordeiro and Hincke [32] who showed the occurrence of fibronectin, vitellogenin, and apolipoproteins in the shell membranes of fertilized eggs. The HESM also contained many different enzymes, protease inhibitors, and signaling proteins similar to fresh ESM. However, the HESM seemed to be differentially populated with several proteins associated with glycolysis, carbohydrate, protein, and nucleic acid metabolisms. Many ribosomal, histone, and ribonuclear proteins, and enzymes, associated with protein folding and translocations, were also identified in the HESM extract. However, there was a conspicuous absence of clusterin, a glycoprotein which we and others have found present in the fresh ESM and has been reported as a major component of egg proteome [17, 19, 21, 33–36]. Clusterin is implicated in the maintenance of cellular homeostasis, developmental remodeling, and in the egg, it is correlated with the changes in viscosity of egg white [36–39]. In our previous study with fresh ESM we identified several clusterin peptide fragments in the methanol extract of ESM. However, the absence of any clusterin peptide in HESM under similar extraction condition was intriguing. It is possible that clusterin, in shell membrane, is involved in early embryo development and utilized completely, as the development reaches to its termination. Similarly, there was also a conspicuous absence of gallin, an antimicrobial peptide that occurs uniquely in egg proteome of fresh eggs [17, 33, 40] whereas two other avian defensins, gallinacin 9 and 10, were identified along with several other antimicrobial proteins including ovotransferrin, lysozyme, keratin, and ovomucoid. Because the protein profiles of egg undergo qualitative and quantitative changes both, during incubation and passive storage [21, 22, 32, 38, 41, 42], the differences in protein profiles of membranes between fresh and hatchery shells appear logical and consistent.

A functional association bioinformatics showed co-occurrence of proteins associated with ubiquitination and apoptosis activities, indicative of large scale protein degradation, expected, with the embryo development and hatching. The other notable protein clusters were carbohydrate and energy metabolizing enzymes (glyceraldehyde phosphate dehydrogenase, α-enolase, isocitrate dehydrogenase, aldolase, triose phosphate isomerase) as well as the proteins associated to nucleic acid metabolism, and stress protection functions such as heat shock and heat shock cognate proteins (HSP). Besides the endogenous proteins and peptides, the HESM also contained several proteins identified with multi species of both gram positive and gram negative bacteria some belonging to families of phytobacteria, soil bacteria even, marine bacteria. Proteins identified with bacteria such as Candidatus, Enterococci, Yersinia, Butyrivibrio Pseudomonas, Clostridium, and Vibrio are zoonotic that are implicated in human gastrointestinal and respiratory illness including one peptide that belonged to a Eimeria, a protozoa, that causes coccidiosis and enteritis in poultry.

The objective of the study was to gain a better understanding of the potential of egg membrane as an adjuvant that likely provides epigenetic conditioning influencing the post hatch immunity through its proteomic constituents [20]. Based on the above results, it appears that the HESM with its plethora of bioactive proteins, peptides, enzymes, and multi species microbial protein factors, may be a suitable modulator of immunity of post hatch chickens with its muco-adhesive properties and action on gastrointestinal mucosa. Besides, the HESM protein profile also provides a reference to compare and identify similar other allogeneic and xenogeneic sources of material that can improve immunity of post hatch poultry reducing the dependence on antibiotics growth promoters. The identification of diverse bacterial proteins in the HESM makes it appealing since numerous body of evidence has shown that the immunity and disease resistance is greatly influenced by biodiverse factors that include various bacterial and viral antigens [43–45].

## Conclusion

The HESM is a rich source of a variety of bioactive proteins particularly belonging to signaling enzymatic, and regulatory varieties that along with many microbial proteins make it uniquely suitable for use as a modulator to improve post hatch immunity of poultry.

## Additional file

Additional file 1: Table S1. Proteins identified in guanidine HCL extract of eggshell membranes by LC-MS/MS. Proteins identified with a single peptide are considered “**tentatively identified**”. **Table S2.** Bacterial proteins identified in Guanidine HCl extracts of HESM. Proteins identified with one only peptide are considered “**tentatively identified**”. (DOCX 68 kb)

## Abbreviations

ACN: Acetonitrile
BCA: Bicinchoninic acid
DTT: Dithiothreitol
ESM: Egg shell membrane
FA: Formic acid
GuHCl: Guanidine hydrochloride
HESM: Hatchery egg shell membrane
IAA: Iodoacetamide
LC-MS/MS: Liquid chromatography-tandem mass spectrometry
MALDI-TOF: Matrix assisted laser desorption ionization time of flight
MS: Mass spectrometry
