# Supplementary material for: Protein profiles of hatchery egg shell membrane
Source: Proteome Sci. 2017 Mar 3;15:4. doi: 10.1186/s12953-017-0112-6 (PMC5335749; doi:10.1186/s12953-017-0112-6)
Supplement: Additional file 1: Table S1. — Proteins identified in guanidine HCL extract of eggshell membranes by LC-MS/MS. Proteins identified with a single peptide are considered “tentatively identified”. Table S2. Bacterial proteins identified in Guanidine HCl extracts of HESM. Proteins identified with one only peptide are considered “tentatively identified”. (DOCX 68 kb) [file 12953_2017_112_MOESM1_ESM.docx]

**Table S1**: Proteins identified in guanidine HCL extract of eggshell membranes by LC-MS/MS. Proteins identified with a single peptide are considered “**tentatively identified**”.

|  | **Protein id** | **Description** | **Score** | **MW** | **# Peptides** |
| --- | --- | --- | --- | --- | --- |
| 1 | ENSGALP00000010405 | Ovotransferrin precursor [Source: RefSeq peptide;Acc:NP_990635] | 77.8 | 2328.2 | 45 |
| 2 | ENSGALP00000036403 | Ovalbumin [Source: UniProtKB/Swiss-Prot;Acc:P01012] | 42.9 | 1751.1 | 28 |
| 3 | ENSGALP00000039176 | Actin, cytoplasmic type 5 [Source: UniProtKB/Swiss-Prot;Acc:P53478] | 41.8 | 1470.9 | 29 |
| 4 | ENSGALP00000005654 | fibronectin precursor [Source: RefSeq peptide;Acc:NP_001185641] | 273.1 | 1383 | 33 |
| 5 | ENSGALP00000019372 | protein-glutamine gamma-glutamyltransferase 4 [Source: RefSeq peptide;Acc:NP_001006368] | 78.9 | 1307.4 | 28 |
| 6 | ENSGALP00000016648 | Uncharacterized | 23.9 | 1267.7 | 17 |
| 7 | ENSGALP00000015988 | Actin, alpha cardiac muscle 1 [Source: UniProtKB/Swiss-Prot;Acc:P68034] | 42 | 1267.2 | 24 |
| 8 | ENSGALP00000006093 | keratin, type I cytoskeletal 19 [Source: RefSeq peptide;Acc:NP_990340] | 46 | 1110.2 | 28 |
| 9 | ENSGALP00000005836 | keratin, type I cytoskeletal 14-like [Source: RefSeq peptide;Acc:NP_001264913] | 50 | 904.6 | 23 |
| 10 | ENSGALP00000016632 | Keratin 8, type II [Source: HGNC Symbol;Acc:HGNC:6446] | 42.1 | 870.4 | 17 |
| 11 | ENSGALP00000006090 | keratin, type I cytoskeletal 14 [Source: RefSeq peptide;Acc:NP_001001311] | 51 | 845.1 | 20 |
| 12 | ENSGALP00000015687 | collagen alpha-2(I) chain precursor [Source: RefSeq peptide;Acc:NP_001073182] | 128.8 | 805.3 | 19 |
| 13 | ENSGALP00000038912 | Gallus gallus alpha-D-globin (HBAD), mRNA. [Source: RefSeq mRNA;Acc:NM_001004375] | 15.7 | 776.9 | 13 |
| 14 | ENSGALP00000005971 | Annexin A2 [Source: UniProtKB/Swiss-Prot;Acc:P17785] | 38.6 | 740.3 | 17 |
| 15 | ENSGALP00000016177 | Lysozyme C [Source: UniProtKB/Swiss-Prot;Acc:P00698] | 16.2 | 712.1 | 11 |
| 16 | ENSGALP00000035593 | Hemoglobin subunit beta [Source: UniProtKB/ Swiss-Prot;Acc:P02112] | 16.5 | 712.1 | 12 |
| 17 | ENSGALP00000016651 | Uncharacterized protein [Source:UniProtKB/TrEMBL;Acc:H9KZP6] | 28 | 701.3 | 16 |
| 18 | ENSGALP00000019031 | serum albumin precursor [Source: RefSeq peptide;Acc:NP_990592] | 64 | 673.2 | 17 |
| 19 | ENSGALP00000038904 | Hemoglobin, alpha 1 (HBAA), mRNA. [Source:RefSeq mRNA;Acc:NM_001004376] | 15.4 | 668.8 | 13 |
| 20 | ENSGALP00000018373 | decorin precursor [Source: RefSeq peptide;Acc:NP_001025918] | 39.6 | 654.7 | 15 |
| 21 | ENSGALP00000014107 | vimentin [Source: RefSeq peptide;Acc:NP_001041541] | 53.2 | 641.7 | 14 |
| 22 | ENSGALP00000035339 | Gallus gallus ATP synthase, H+ transporting, mitochondrial F1 complex, beta polypeptide (ATP5B), nuclear gene encoding mitochondrial protein, mRNA. [Source: efSeq mRNA;Acc:NM_001031391] | 52.9 | 538.6 | 10 |
| 23 | ENSGALP00000035591 | epsilon globin [Source: RefSeq peptide;Acc:NP_001026660] | 16.4 | 522.6 | 11 |
| 24 | ENSGALP00000006098 | keratin, type I cytoskeletal 15 [Source: RefSeq peptide;Acc:NP_001001312] | 47.9 | 486.5 | 15 |
| 25 | ENSGALP00000043135 | titin [Source: HGNC Symbol;Acc:HGNC:12403] | 3397.7 | 456.8 | 27 |
| 26 | ENSGALP00000041526 | Gallus gallus histone cluster 1, H4-VI, germinal H4 (similar to human histone cluster 1, class H4 genes) (HIST1H46), mRNA. [Source: RefSeq mRNA;Acc:NM_001037845] | 11.4 | 449 | 6 |
| 27 | ENSGALP00000022528 | zona pellucida sperm-binding protein 1 precursor [Source: RefSeq peptide;Acc:NP_990014] | 99.7 | 443.2 | 10 |
| 28 | ENSGALP00000003737 | alpha-enolase [Source: RefSeq peptide;Acc:NP_990451] | 47.3 | 397.4 | 8 |
| 29 | ENSGALP00000003695 | keratin 6A [Source: RefSeq peptide;Acc:NP_001001313] | 57 | 397 | 9 |
| 30 | ENSGALP00000038799 | Tropomyosin alpha-1 chain [Source: UniProtKB/Swiss-Prot;Acc:P04268] | 32.9 | 392.9 | 12 |
| 31 | ENSGALP00000010210 | myeloid protein 1 precursor [Source: RefSeq peptide;Acc:NP_990809] | 35.2 | 388.4 | 7 |
| 32 | ENSGALP00000042171 | transgelin 2 [Source: HGNC Symbol;Acc:HGNC:11554] | 29 | 382 | 8 |
| 33 | ENSGALP00000023278 | Glyceraldehyde-3-phosphate dehydrogenase [Source: UniProtKB/Swiss-Prot;Acc:P00356] | 34.9 | 366.1 | 8 |
| 34 | ENSGALP00000032184 | Annexin A1 [Source: RefSeq peptide;Acc:NP_996789] | 38.5 | 334 | 9 |
| 35 | ENSGALP00000002368 | Zona pellucida sperm-binding protein 3 Processed zona pellucida sperm-binding protein 3 [Source: UniProtKB/Swiss-Prot;Acc:P79762] | 46.7 | 331.6 | 3 |
| 36 | ENSGALP00000025606 | Elongation factor 1-alpha 1 [Source: UniProtKB/Swiss-Prot;Acc:Q90835] | 50.1 | 328.2 | 10 |
| 37 | ENSGALP00000037266 | Gallus gallus histone cluster 1, H1.01 (similar to human histone cluster 1, class H1 genes) (HIST1H101), mRNA. [Source: RefSeq mRNA;Acc:NM_001040642] | 22.5 | 326.7 | 7 |
| 38 | ENSGALP00000041690 | peptidyl-prolyl cis-trans isomerase A [Source: RefSeq peptide;Acc:NP_001159798] | 15.9 | 323.2 | 6 |
| 39 | ENSGALP00000011510 | Apolipoprotein A-I Proapolipoprotein A-I [Source: UniProtKB/Swiss-Prot;Acc:P08250] | 30.7 | 320.7 | 8 |
| 40 | ENSGALP00000042590 | keratin 18, type I [Source:HGNC Symbol;Acc:HGNC:6430] | 19 | 318.3 | 5 |
| 41 | ENSGALP00000018370 | Lumican [Source: UniProtKB/Swiss-Prot;Acc:P51890] | 38.6 | 317 | 8 |
| 42 | ENSGALP00000009563 | Annexin [Source: UniProtKB/TrEMBL;Acc:E1C8K3] | 36.7 | 313.8 | 8 |
| 43 | ENSGALP00000017755 | Ovocleidin-116 [Source: UniProtKB/Swiss-Prot;Acc:F1NSM7] | 76.8 | 312.2 | 6 |
| 44 | ENSGALP00000019120 | Heat shock 70 kDa protein [Source: RefSeq peptide;Acc:NP_001006686] | 69.9 | 300.9 | 11 |
| 45 | ENSGALP00000013964 | tubulin beta-3 chain [Source: RefSeq peptide;Acc:NP_001074329] | 49.8 | 272.9 | 7 |
| 46 | ENSGALP00000002197 | gelsolin precursor [Source: RefSeq peptide;Acc:NP_990265] | 85.8 | 272.4 | 10 |
| 47 | ENSGALP00000005544 | Ovomucoid [Source: UniProtKB/Swiss-Prot;Acc:P01005] | 22.6 | 269.7 | 5 |
| 48 | ENSGALP00000018265 | Serpin H1 [Source: UniProtKB/Swiss-Prot;Acc:P13731] | 45.7 | 264.9 | 1 |
| 49 | ENSGALP00000043256 | Fructose-bisphosphate aldolase C [Source: UniProtKB/Swiss-Prot;Acc:P53449] | 39.3 | 257.2 | 7 |
| 50 | ENSGALP00000028845 | keratin 4, type II [Source:HGNC Symbol;Acc:HGNC:6441] | 58.7 | 256.4 | 8 |
| 51 | ENSGALP00000010510 | heat shock cognate 71 kDa protein [Source: RefSeq peptide;Acc:NP_990334] | 70.8 | 255.4 | 2 |
| 52 | ENSGALP00000023396 | Triosephosphate isomerase [Source: UniProtKB/Swiss-Prot;Acc:P00940] | 26.6 | 231.3 | 4 |
| 53 | ENSGALP00000023085 | Gallus gallus actinin, alpha 4 (ACTN4), mRNA. [Source :RefSeq mRNA;Acc:NM_205126] | 71.6 | 217.2 | 5 |
| 54 | ENSGALP00000018742 | creatine kinase B-type [Source: RefSeq peptide;Acc:NP_990641] | 40.2 | 216.4 | 8 |
| 55 | ENSGALP00000005607 | proline/arginine-rich end leucine-rich repeat protein [Source: HGNC Symbol;Acc:HGNC:9357] | 42.9 | 210.9 | 6 |
| 56 | ENSGALP00000018424 | desmin [Source: HGNC Symbol;Acc:HGNC:2770] | 48.8 | 204.5 | 6 |
| 57 | ENSGALP00000034108 | Pyruvate kinase PKM [Source: UniProtKB/Swiss-Prot;Acc:P00548] | 57.8 | 200.1 | 6 |
| 58 | ENSGALP00000021743 | Histone H2B [Source: UniProtKB/TrEMBL;Acc:F1NF30] | 13.5 | 193 | 1 |
| 59 | ENSGALP00000014097 | destrin [Source: RefSeq peptide;Acc:NP_990859] | 18.4 | 189.9 | 7 |
| 60 | ENSGALP00000023926 | Uncharacterized protein [Source: UniProtKB/TrEMBL;Acc:E1C6R9] | 19.9 | 182.5 | 4 |
| 61 | ENSGALP00000020275 | Beta-galactoside-binding lectin [Source: UniProtKB/Swiss-Prot;Acc:P07583] | 15.1 | 176.9 | 2 |
| 62 | ENSGALP00000017578 | anterior gradient 2 [Source: HGNC Symbol;Acc:HGNC:328] | 19.8 | 173.9 | 4 |
| 63 | ENSGALP00000039133 | 14-3-3 protein epsilon [Source: UniProtKB/Swiss-Prot;Acc:Q5ZMT0] | 26.6 | 173.4 | 3 |
| 64 | ENSGALP00000025593 | collagen alpha-1(XII) chain precursor [Source: RefSeq peptide;Acc:NP_990352] | 339.6 | 171.6 | 1 |
| 65 | ENSGALP00000010853 | Uncharacterized protein | 452.4 | 161.2 | 3 |
| 66 | ENSGALP00000042357 | H2A histone family, member X [Source: HGNC Symbol;Acc:HGNC:4739] | 15 | 159.5 | 3 |
| 67 | ENSGALP00000042528 | cold-inducible RNA-binding protein [Source: RefSeq peptide;Acc:NP_001026518] | 18..6 | 159.5 | 2 |
| 68 | ENSGALP00000008163 | orsomucoid 1 (ovoglycoprotein) precursor [Source: RefSeq peptide;Acc:NP_989872] | 22.3 | 158.4 | 3 |
| 69 | ENSGALP00000018498 | heat shock protein 90kDa alpha (cytosolic), class A member 1 [Source: HGNC Symbol;Acc:HGNC:5253] | 83.2 | 157.7 | 3 |
| 70 | ENSGALP00000041937 | tropomyosin beta chain [Source: RefSeq peptide;Acc:NP_990777] | 28.7 | 157.6 | 7 |
| 71 | ENSGALP00000001474 | 78 kDa glucose-regulated protein [Source: UniProtKB/Swiss-Prot;Acc:Q90593] | 72 | 155.8 | 5 |
| 72 | ENSGALP00000025280 | Thioredoxin [Source: UniProtKB/Swiss-Prot;Acc:P08629] | 11.7 | 155.4 | 4 |
| 73 | ENSGALP00000006240 | collagen alpha-3(VI) chain precursor [Source:RefSeq peptide;Acc:NP_990865] | 339.4 | 155.2 | 5 |
| 74 | ENSGALP00000019399 | Transgelin [Source: UniProtKB/Swiss-Prot;Acc:P19966] | 22.3 | 147.2 | 5 |
| 75 | ENSGALP00000033366 | Ribonuclease homolog [Source: UniProtKB/Swiss-Prot;Acc:P30374] | 15.9 | 143.2 | 1 |
| 76 | ENSGALP00000020967 | ovalbumin-related protein Y [Source: RefSeq peptide;Acc:NP_001026172] | 43.8 | 141.6 | 5 |
| 77 | ENSGALP00000028277 | Histone H2A.Z [Source: UniProtKB/Swiss-Prot;Acc:Q5ZMD6] | 13.6 | 139.7 | 3 |
| 78 | ENSGALP00000040188 | S100 calcium binding protein A12 [Source: HGNC Symbol;Acc:HGNC:10489] | 18.5 | 138.4 | 4 |
| 79 | ENSGALP00000036122 | 14-3-3 protein theta [Source: UniProtKB/Swiss-Prot;Acc:Q5ZMD1] | 27.8 | 138.3 | 1 |
| 80 | ENSGALP00000002888 | vitellogenin-2 precursor [Source: RefSeq peptide;Acc:NP_001026447] | 205 | 136.8 | 6 |
| 81 | ENSGALP00000019033 | Alpha-fetoprotein [Source: UniProtKB/TrEMBL;Acc:E1BV96] | 71.1 | 133.9 | 6 |
| 82 | ENSGALP00000040672 | stratifin [Source: HGNC Symbol;Acc:HGNC:10773] | 27.7 | 133.4 | 4 |
| 83 | ENSGALP00000010852 | Uncharacterized protein [Source: UniProtKB/TrEMBL;Acc:F1NZY2] | 233.4 | 133.1 | 2 |
| 84 | ENSGALP00000026846 | Gallinacin-10 [Source: UniProtKB/Swiss-Prot;Acc:Q6QLQ9] | 7.1 | 127.7 | 2 |
| 85 | ENSGALP00000043172 | Cystatin [Source: UniProtKB/Swiss-Prot;Acc:P01038] | 16.3 | 123.6 | 2 |
| 86 | ENSGALP00000041913 | Myosin regulatory light chain 2, smooth muscle major isoform [Source:UniProtKB/Swiss-Prot;Acc:P02612] | 19.8 | 117.8 | 4 |
| 87 | ENSGALP00000000876 | fatty acid-binding protein, heart [Source: RefSeq peptide;Acc:NP_001026060] | 14.8 | 116.4 | 4 |
| 88 | ENSGALP00000038626 | L-lactate dehydrogenase A chain [Source: RefSeq peptide;Acc:NP_990615] | 36.5 | 116.2 | 4 |
| 89 | ENSGALP00000011717 | 60S acidic ribosomal protein P0 [Source:RefSeq peptide;Acc:NP_990318] | 34.6 | 114.8 | 2 |
| 90 | ENSGALP00000011961 | phosphatidylethanolamine-binding protein 1 [Source: RefSeq peptide;Acc:NP_001185571] | 20.9 | 113.9 | 4 |
| 91 | ENSGALP00000000062 | Uncharacterized protein [Source: UniProtKB/TrEMBL;Acc:H9KYP2] | 67.8 | 113.8 | 5 |
| 92 | ENSGALP00000043361 | Uncharacterized protein [Source: UniProtKB/TrEMBL;Acc:R4GMA5] | 26.7 | 113.2 | 2 |
| 93 | ENSGALP00000043060 | Mimecan [Source: UniProtKB/Swiss-Prot;Acc:Q9W6H0] | 33.2 | 110.1 | 3 |
| 94 | ENSGALP00000013267 | rab GDP dissociation inhibitor beta [Source: RefSeq peptide;Acc:NP_990335] (Osteoglycine) | 50.7 | 106.7 | 3 |
| 95 | ENSGALP00000034078 | Nucleoside diphosphate kinase [Source: UniProtKB/Swiss-Prot;Acc:O57535] | 17.3 | 104 | 4 |
| 96 | ENSGALP00000019365 | annexin A5 [Source: RefSeq peptide;Acc:NP_001026709] | 36.2 | 103.3 | 5 |
| 97 | ENSGALP00000026126 | 60S ribosomal protein L8 [Source: RefSeq peptide;Acc:NP_001264657] | 28 | 102.2 | 4 |
| 98 | ENSGALP00000005520 | aldehyde dehydrogenase 9 family, member A1 [Source: HGNC Symbol;Acc:HGNC:412] | 57.1 | 101.7 | 3 |
| 99 | ENSGALP00000000316 | major vault protein [Source: RefSeq peptide;Acc:NP_001006336] | 93.7 | 96.5 | 6 |
| 100 | ENSGALP00000036963 | ribosomal protein L15 [Source: HGNC Symbol;Acc:HGNC:10306] | 24.1 | 95.8 | 2 |
| 101 | ENSGALP00000020094 | Histone H5 [Source: UniProtKB/Swiss-Prot;Acc:P02259] | 20.7 | 95.4 | 3 |
| 101 | ENSGALP00000014317 | isocitrate dehydrogenase 1 (NADP+), soluble [Source: HGNC Symbol;Acc:HGNC:5382] | 46.9 | 93.3 | 2 |
| 102 | ENSGALP00000008498 | transketolase [Source:HGNC Symbol;Acc:HGNC:11834] | 68.4 | 91.9 | 3 |
| 103 | ENSGALP00000039326 | 60S ribosomal protein L19 [Source: RefSeq peptide;Acc:NP_001026100] | 23.2 | 85.7 | 2 |
| 104 | ENSGALP00000014912 | Protein S100-A11 [Source: UniProtKB/Swiss-Prot;Acc:P24479] | 11.4 | 85 | 1 |
| 105 | ENSGALP00000013574 | Protein disulfide-isomerase A3 [Source: UniProtKB/Swiss-Prot;Acc:Q8JG64] | 56.1 | 84.9 | 4 |
| 106 | ENSGALP00000016361 | ribosomal protein S3A [Source:RefSeq peptide;Acc:NP_001075886] | 29.8 | 84.7 | 4 |
| 107 | ENSGALP00000040606 | CD99 antigen precursor [Source: RefSeq peptide;Acc:NP_001185580] | 18.2 | 79.3 | 2 |
| 108 | ENSGALP00000006938 | annexin A6 [Source: RefSeq peptide;Acc:NP_990061] | 75.2 | 77.5 | 2 |
| 109 | ENSGALP00000040966 | Uncharacterized protein [Source: UniProtKB/TrEMBL;Acc:R4GG07] | 13.2 | 74.1 | 1 |
| 110 | ENSGALP00000007490 | moesin [Source: HGNC Symbol;Acc:HGNC:7373] | 68.5 | 73.8 | 3 |
| 111 | ENSGALP00000038677 | Gallus gallus phosphoglycerate mutase 1 (brain) (PGAM1), mRNA. [Source: RefSeq mRNA;Acc:NM_001031556] | 23.7 | 73.4 | 3 |
| 112 | ENSGALP00000029440 | Vitelline membrane outer layer protein 1 [Source: UniProtKB/Swiss-Prot;Acc:P41366] | 20.2 | 69.1 | 1 |
| 113 | ENSGALP00000024468 | 40S ribosomal protein S15 [Source: UniProtKB/Swiss-Prot;Acc:P62846] | 16.9 | 68 | 2 |
| 114 | ENSGALP00000032611 | vitellogenin-1 precursor [Source: RefSeq peptide;Acc:NP_001004408] | 210.6 | 67.6 | 3 |
| 115 | ENSGALP00000003455 | peptidyl-prolyl cis-trans isomerase B precursor [Source: RefSeq peptide;Acc:NP_990792] | 16.2 | 66.6 | 2 |
| 116 | ENSGALP00000029968 | 60S ribosomal protein L3 [Source: RefSeq peptide;Acc:NP_001006241] | 26.1 | 65.8 | 3 |
| 117 | ENSGALP00000025929 | hydroxyacyl-coenzyme A dehydrogenase, mitochondrial [Source: RefSeq peptide;Acc:NP_001264826] | 34.4 | 64.9 | 2 |
| 118 | ENSGALP00000001013 | 60S ribosomal protein L22 [Source: RefSeq peptide;Acc:NP_989472] | 14.6 | 64.7 | 2 |
| 119 | ENSGALP00000007680 | 60S ribosomal protein L6 [Source: RefSeq peptide;Acc:NP_989483] | 33.9 | 64 | 2 |
| 120 | ENSGALP00000021618 | L-lactate dehydrogenase B chain [Source: UniProtKB/Swiss-Prot;Acc:P00337] | 36.3 | 61.6 | 3 |
| 121 | ENSGALP00000038462 | protein TENP [Source: RefSeq peptide;Acc:NP_990357] | 47.4 | 61.3 | 2 |
| 122 | ENSGALP00000011689 | protein disulfide-isomerase precursor [Source: RefSeq peptide;Acc:NP_001185639] | 55.8 | 61.1 | 4 |
| 123 | ENSGALP00000003431 | nucleophosmin [Source: RefSeq peptide;Acc:NP_990598] | 30.3 | 60.5 | 2 |
| 124 | ENSGALP00000041639 | ribosomal protein S26 [Source: HGNC Symbol;Acc:HGNC:10414] | 18 | 59 | 2 |
| 125 | ENSGALP00000008131 | vinculin [Source: HGNC Symbol;Acc:HGNC:12665] | 114.3 | 58.9 | 1 |
| 126 | ENSGALP00000024078 | WD repeat-containing protein 1 [Source: RefSeq peptide;Acc:NP_001004402] | 66.5 | 58.5 | 1 |
| 127 | ENSGALP00000023089 | ribosomal protein, large, P2 [Source: HGNC Symbol;Acc:HGNC:10377] | 14.2 | 57.8 | 1 |
| 128 | ENSGALP00000007476 | protein SET [Source: RefSeq peptide;Acc:NP_001025862] | 32.1 | 57.7 | 1 |
| 129 | ENSGALP00000012462 | ribosomal protein S25 [Source: HGNC Symbol;Acc:HGNC:10413] | 13.7 | 57.5 | 1 |
| 130 | ENSGALP00000000509 | heterogeneous nuclear ribonucleoprotein M [Source: RefSeq peptide;Acc:NP_001026103] | 76 | 53 | 4 |
| 131 | ENSGALP00000014298 | 60S ribosomal protein L12 [Source: RefSeq peptide;Acc:NP_001264608] | 17.7 | 52.2 | 1 |
| 132 | ENSGALP00000027030 | 60S ribosomal protein L31 [Source: RefSeq peptide;Acc:NP_001264684] | 14.7 | 52.2 | 2 |
| 133 | ENSGALP00000027665 | acetyl-CoA acetyltransferase, mitochondrial [Source: RefSeq peptide;Acc:NP_001264708] | 44.1 | 51.9 | 2 |
| 134 | ENSGALP00000039447 | heat shock protein beta-1 [Source: RefSeq peptide;Acc:NP_990621] | 21.8 | 51.9 | 1 |
| 135 | ENSGALP00000025745 | Uncharacterized protein [Source: UniProtKB/TrEMBL;Acc:F1P304] | 24.5 | 50.8 | 3 |
| 136 | ENSGALP00000039530 | heterogeneous nuclear ribonucleoprotein H [Source: RefSeq peptide;Acc:NP_989827] | 56.5 | 50.4 | 2 |
| 137 | ENSGALP00000038435 | protein-glutamine gamma-glutamyltransferase 2 [Source: RefSeq peptide;Acc:NP_990779] | 77.7 | 49.5 | 1 |
| 138 | ENSGALP00000002333 | Uncharacterized protein [Source: UniProtKB/TrEMBL;Acc:F1NPG6] | 93.8 | 47.6 | 2 |
| 139 | ENSGALP00000006284 | heterogeneous nuclear ribonucleoprotein H3 [Source: RefSeq peptide;Acc:NP_001012610] | 36.6 | 46.3 | 2 |
| 140 | ENSGALP00000021314 | caldesmon [Source: RefSeq peptide;Acc:NP_989489] | 86.4 | 44.7 | 2 |
| 141 | ENSGALP00000026392 | ribosomal protein S7 [Source:HGNC Symbol;Acc:HGNC:10440] | 22.3 | 43 | 2 |
| 142 | ENSGALP00000026123 | ATP-dependent RNA helicase DDX3X [Source: RefSeq peptide;Acc:NP_001025971] | 72.6 | 42.8 | 1 |
| 143 | ENSGALP00000027012 | lysozyme g precursor [Source: RefSeq peptide;Acc:NP_001001470] | 23.3 | 42.2 | 3 |
| 144 | ENSGALP00000019979 | Uncharacterized protein [Source: UniProtKB/TrEMBL;Acc:F1NI80] | 41.1 | 41.2 | 1 |
| 145 | ENSGALP00000010800 | actin-related protein 2/3 complex subunit 4 [Source :RefSeq peptide;Acc:NP_001244213] | 19.7 | 41.1 | 8 |
| 146 | ENSGALP00000025525 | Carbonic anhydrase 2 [Source: UniProtKB/Swiss-Prot;Acc:P07630] | 29 | 41.1 | 1 |
| 147 | ENSGALP00000039575 | guanine nucleotide-binding protein G(I)/G(S)/G(T) subunit beta-1 [Source: RefSeq peptide;Acc:NP_001012853] | 37.3 | 40.8 | 2 |
| 148 | ENSGALP00000028211 | glycerol-3-phosphate dehydrogenase 1 (soluble) [Source: HGNC Symbol;Acc:HGNC:4455] | 38.6 | 40.2 | 1 |
| 149 | ENSGALP00000014746 | Gallus gallus heat shock 10kDa protein 1 (chaperonin 10) (HSPE1), nuclear gene encoding mitochondrial protein, mRNA. [Source: RefSeq mRNA;Acc:NM_205067] | 12.1 | 39.6 | 2 |
| 150 | ENSGALP00000035996 | superoxide dismutase [Source: RefSeq peptide;Acc:NP_990395] | 15.7 | 39.3 | 1 |
| 151 | ENSGALP00000012481 | 60S ribosomal protein L4 [Source: RefSeq peptide;Acc:NP_001007480] | 46.7 | 38.6 | 2 |
| 152 | ENSGALP00000006308 | ribosomal protein L23a [Source:HGNC Symbol;Acc:HGNC:10317] | 17.6 | 38.3 | 2 |
| 153 | ENSGALP00000010414 | heterogeneous nuclear ribonucleoprotein G [Source: RefSeq peptide;Acc:NP_001073196] | 41.5 | 38 | 2 |
| 154 | ENSGALP00000001914 | chloride intracellular channel 4 [Source: HGNC Symbol;Acc:HGNC:13518] | 27.7 | 31.5 | 1 |
| 155 | ENSGALP00000041423 | glutathione peroxidase 1 [Source:RefSeq peptide;Acc:NP_001264782] | 17.9 | 31.4 | 1 |
| 156 | ENSGALP00000008802 | 40S ribosomal protein S2 [Source: RefSeq peptide;Acc:NP_001264093] | 30.7 | 31 | 1 |
| 157 | ENSGALP00000013122 | 60 kDa heat shock protein, mitochondrial [Source: UniProtKB/Swiss-Prot;Acc:Q5ZL72] | 60.9 | 30.8 | 1 |
| 158 | ENSGALP00000009511 | 60S ribosomal protein L5 [Source:UniProtKB/Swiss-Prot;Acc:P22451] | 33.9 | 30.7 | 2 |
| 159 | ENSGALP00000006222 | calreticulin 3 [Source:HGNC Symbol;Acc:HGNC:20407] | 48 | 29.8 | 1 |
| 160 | ENSGALP00000041109 | capping protein (actin filament), gelsolin-like [Source:HGNC Symbol;Acc:HGNC:1474] | 72.4 | 29.5 | 1 |
| 161 | ENSGALP00000033411 | ribosomal protein S21 [Source:HGNC Symbol;Acc:HGNC:10409] | 9.1 | 28.8 | 1 |
| 162 | ENSGALP00000029993 | rho GDP-dissociation inhibitor 2 [Source: RefSeq peptide;Acc:NP_001264293] | 23.2 | 28.1 | 1 |
| 163 | ENSGALP00000035366 | isocitrate dehydrogenase [Source: RefSeq peptide;Acc:NP_001026770] | 50.4 | 26.7 | 1 |
| 164 | ENSGALP00000035959 | carbonyl reductase [Source:RefSeq peptide;Acc:NP_001025966] | 30.3 | 24.4 | 1 |
| 165 | ENSGALP00000041772 | Protein syndesmos [Source:UniProtKB/Swiss-Prot;Acc:Q9IAY5] | 33.8 | 23.5 | 1 |
| 166 | ENSGALP00000003373 | 40S ribosomal protein S17 [Source:UniProtKB/Swiss-Prot;Acc:P08636] | 9.5 | 20 | 1 |
| 167 | ENSGALP00000037222 | Heterogeneous nuclear ribonucleoprotein D-like [Source: UniProtKB/Swiss-Prot;Acc:Q5ZI72] | 33.4 | 17.3 | 1 |

**Table S**2: Bacterial proteins identified in Guanidine HCl extracts of HESM. Proteins identified with one only peptide are considered “**tentatively identified**”.

|  | **Accession #** | **Protein** | **MW [kDa]** | **Scores** | **#Peptides** |
| --- | --- | --- | --- | --- | --- |
| 1 | gi\|294828133 | histidine kinase/response regulator hybrid protein [**Leptospira interrogans serovar Lai str. 56601**] | 116.9 | 77.8 | 4 |
| 2 | gi\|517357534 | serine/threonine protein kinase [**Streptomyces sp. HmicA12]** | 67.9 | 65.8 | 2 |
| 3 | gi\|655245428 | protein kinase [**Nocardioides sp. J54**] | 72.9 | 63.7 | 2 |
| 4 | gi\|124514345 | Precorrin-4 C11-methyltransferase [**Leptospirillum rubarum**] | 29.5 | 56.8 | 2 |
| 5 | gi\|206742631 | soluble lytic murein transglycosylase [**Thermodesulfovibrio yellowstonii DSM 11347**] | 73.8 | 55.6 | 2 |
| 6 | gi\|292642245 | SWIM zinc finger domain protein [**Enterococcus faecium PC4.1]** | 42.4 | 54.1 | 2 |
| 7 | gi\|695172258 | aminotransferase [**Sphingomonas taxi**] | 33.6 | 49.5 | 1 |
| 8 | gi\|345633807 | LuxR family transcriptional regulator [**Streptomyces zinciresistens K42**] | 24.0 | 48.9 | 1 |
| 9 | gi\|19705092 | hypothetical protein FN1787 [**Fusobacterium nucleatum subsp. nucleatum ATCC 25586**] | 73.8 | 44.1 | 1 |
| 10 | gi\|124515012 | putative hydrolase, haloacid dehalogenase-like family [**Leptospirillum rubarum**] | 24.5 | 43.7 | 1 |
| 11 | gi\|422886479 | cold shock-like protein [**Alcaligenes sp. HPC1271**] | 7.5 | 42.3 | 1 |
| 12 | gi\|618782811 | ribonucleotide-diphosphate reductase subunit alpha [**Pseudomonas aeruginosa M10**] | 107.0 | 38.8 | 1 |
| 13 | gi\|311693188 | ATPase components of ABC transporters with duplicated ATPase domains [**Marinobacter adhaerens HP15**] | 24.5 | 71.7 | 2 |
| 14 | gi\|297550774 | serine/threonine protein kinase with TPR repeats [**Ktedonobacter racemifer DSM 44963**] | 73.8 | 65 | 2 |
| 15 | gi\|405587154 | transcription termination factor Rho [**Bergeyella zoohelcum CCUG 30536]** | 65.9 | 63.3 | 2 |
| 16 | gi\|618771208 | F0F1 ATP synthase subunit alpha [**Pseudomonas aeruginosa M10]** | 55.4 | 61 | 1 |
| 17 | gi\|588290902 | prolyl-tRNA synthetase [**Thalassolituus oleivorans R6-15]** | 63.9 | 55.7 | 2 |
| 18 | gi\|114739665 | isocitrate dehydrogenase, NADP-dependent [**Hyphomonas neptunium ATCC 15444**] | 45.6 | 52.6 | 1 |
| 19 | gi\|755437351 | der GTPase activator family protein [**Yersinia kristensenii]** | 21.2 | 49.5 | 1 |
| 20 | gi\|394456251 | hypothetical protein O71_08395 [**Pontibacter sp. BAB1700]** | 45.3 | 45.8 | 1 |
| 21 | gi\|452006359 | mutant NtrC-like activator [**Pseudomonas stutzeri NF13]** | 51.1 | 45 | 1 |
| 22 | gi\|618789844 | oxidoreductase [**Pseudomonas aeruginosa M10**] | 26.2 | 44.4 | 1 |
| 23 | gi\|328474119 | GTP-binding protein LepA [**Vibrio parahaemolyticus 10329**] | 65.9 | 43.1 | 1 |
| 24 | gi\|651910070 | hypothetical protein [**Butyrivibrio sp. AC2005**] | 52.1 | 40.4 | 1 |
| 25 | gi\|311694265 | glutathione synthase/ribosomal protein S6 modification enzyme [**Marinobacter adhaerens HP15]** | 58.3 | 43.8 | 1 |
| 26 | gi\|546198376 | MULTISPECIES: ribosomal protein L25, Ctc-form [**Bacteria**] | 20.7 | 43.8 | 1 |
| 27 | gi\|726045696 | acetyltransferase [**Candidatus Scalindua brodae**] | 17.6 | 43.4 | 1 |
| 28 | gi\|452009578 | hypothetical protein B381_02321 [**Pseudomonas stutzeri NF13**] | 32.3 | 32.1 | 1 |
| 29 | gi\|258592528 | putative Histidine kinase [**Candidatus Methylomirabilis oxyfera]** | 86.4 | 85.2 | 3 |
| 30 | gi\|516628378 | MULTISPECIES: F0F1 ATP synthase subunit alpha [**Bacteria][Archaea**] | 55.6 | 84.4 | 4 |
| 31 | gi\|292642035 | hypothetical protein CUO_2557 [**Enterococcus faecium PC4.1]** | 97.6 | 80.2 | 3 |
| 32 | gi\|695170760 | 2-keto-4-pentenoate hydratase [**Sphingomonas taxi]** | 35.8 | 78.4 | 3 |
| 33 | gi\|380733894 | serine/threonine protein kinase [**Corallococcus coralloides DSM 2259]** | 126.9 | 77 | 3 |
| 34 | gi\|292637908 | NADH:ubiquinone oxidoreductase, Na(+)-translocating, A subunit [**Bacteroides xylanisolvens SD CC 2a]** | 55.7 | 70.5 | 2 |
| 35 | gi\|114737610 | putative helicase [**Hyphomonas neptunium ATCC 15444]** | 42.9 | 69.2 | 3 |
| 36 | gi\|288328957 | tetratricopeptide repeat protein [**Prevotella sp. oral taxon 317 str. F0108]** | 67.0 | 62.7 | 2 |
| 37 | gi\|618792792 | selenocysteine synthase [**Pseudomonas aeruginosa M10]** | 49.7 | 62 | 2 |
| 38 | gi\|300402166 | DNA mismatch repair domain protein [**Escherichia coli MS 84-1]** | 67.9 | 62 | 2 |
| 39 | gi\|394454349 | tex-like protein [**Pontibacter sp. BAB1700**] | 83.4 | 54.4 | 1 |
| 40 | gi\|695170101 | phosphoadenosine phosphosulfate reductase [**Sphingomonas taxi**] | 27.7 | 51.1 | 1 |
| 41 | gi\|618777703 | ATP-binding protein [**Pseudomonas aeruginosa M10]** | 38.4 | 49.7 | 1 |
| 42 | gi\|291518125 | Uncharacterized protein conserved in bacteria [**Butyrivibrio fibrisolvens 16/4]** | 9.1 | 46.6 | 1 |
| 43 | gi\|726045751 | hypothetical protein SCABRO_01635 [**Candidatus Scalindua brodae]** | 23.3 | 46.2 | 1 |
| 44 | gi\|292643035 | ribonuclease HIII [**Enterococcus faecium PC4.1]** | 33.9 | 45.2 | 1 |
| 45 | gi\|618771210 | F0F1 ATP synthase subunit beta [**Pseudomonas aeruginosa M10]** | 49.5 | 45 | 1 |
| 46 | gi\|691636805 | hypothetical protein IA69_10970 [**Massilia sp. JS1662]** | 99.2 | 44.7 | 1 |
| 47 | gi\|726045041 | hypothetical protein SCABRO_02256 [**Candidatus Scalindua brodae]** | 15.4 | 44.6 | 1 |
| 48 | gi\|635597237 | uncharacterized protein conserved in bacteria [**Comamonadaceae bacterium B1**] | 45.0 | 43.7 | 1 |
| 49 | gi\|114740197 | putative fimbrial assembly protein [**Hyphomonas neptunium ATCC 15444]** | 26.7 | 42.5 | 1 |
| 50 | gi\|667096584 | lytic transglycosylase [**Xanthomonas vasicola pv. vasculorum NCPPB 895]** | 20.7 | 41 | 1 |
